# Health policy triangle framework: Narrative review of the recent literature

**DOI:** 10.1016/j.hpopen.2020.100016

**Published:** 2020-10-06

**Authors:** Gary L. O'Brien, Sarah-Jo Sinnott, Valerie Walshe, Mark Mulcahy, Stephen Byrne

**Affiliations:** a Pharmaceutical Care Research Group, School of Pharmacy, University College Cork, College Road, Cork, Ireland; b Sanofi Genzyme, Cambridge, MA, United States of America; c National Finance Division, Health Service Executive, Model Business Park, Model Farm Road, Cork, Ireland; d Department of Accounting and Finance, Cork University Business School, University College Cork, Cork, Ireland

**Keywords:** Health policy, Policy analysis, Health policy framework, Policy triangle model, Literature review

## Abstract

**Background:**

Developed in the late 20th century, the health policy triangle (HPT) is a policy analysis framework used and applied ubiquitously in the literature to analyse a large number of health-related issues.

**Objective:**

To explore and summarise the application of the HPT framework to health-related (public) policy decisions in the recent literature.

**Methods:**

This narrative review consisted of a systematic search and summary of included articles from January 2015 January 2020. Six electronic databases were searched. Included studies were required to use the HPT framework as part of their policy analysis. Data were analysed using principles of thematic analysis.

**Results:**

Of the 2217 studies which were screened for inclusion, the final review comprised of 54 studies, mostly qualitative in nature. Five descriptive categorised themes emerged (i) health human resources, services and systems, (ii) communicable and non-communicable diseases, (iii) physical and mental health, (iv) antenatal and postnatal care and (v) miscellaneous. Most studies were conducted in lower to upper-middle income countries.

**Conclusion:**

This review identified that the types of health policies analysed were almost all positioned at national or international level and primarily concerned public health issues. Given its generalisable nature, future research that applies the HPT framework to smaller scale health policy decisions investigated at local and regional levels, could be beneficial.

## Highlights

• In 1994, the health policy triangle was first described in the literature.
• Its generalisable nature allows for analysis of many diverse health-related topics.
• In recent years, its utilisation in low and middle-income countries has increased.

## 1 Introduction

The World Health Organisation (WHO) defines health policy as ‘*the decisions, plans, and actions (and inactions) undertaken to achieve specific health care goals within a society or undertaken by a set of institutions and organisations, at national, state and local level, to advance the public's health*’ [1]. Health policy informs decisions like which health technologies to develop and utilise, how to structure and fund health services, and which pharmaceuticals will be freely available [2]. Appreciating the intrinsic relationship between health policy and health, and the impact that other policies have on health, is crucial as it can help to address some of the major health problems that exist. However, health policy decisions are not always the result of a rational process of discussion and evaluation of how a particular objective should be met. The context in which the decisions are made can often be highly political and concern the degree of public provision of healthcare and who pays for it [3]. Health policy decisions can also be conditional on the value judgements implicit in society. As a result, health policies do not always achieve their aims and implementation targets [4,5]. Consequently, health policy analysis is regularly undertaken to understand past policy failures and successes and to plan for future policy implementation [6].

Just as there are various definitions of what policy is, there too are many ideas about the analysis of health policy, and its focus [2,6]. However, what a lot of health policy analysis studies have in common, whether that be analysis *of* policy or analysis *for* policy [7], is the use of a policy framework. A myriad of policy frameworks and theories exists [6]. The burgeoning literature of health policy analysis sees novel policy frameworks being developed quite frequently with the ‘*policy cube*’ approach being the latest addition [8]. A recent literature review investigated the application of some of the more commonly applied frameworks [9]: the advocacy coalition framework (ACF) [10], the stages heuristic model [11], the Kingdon's multiple stream theory [12], the punctuated equilibrium framework [13] and the institutional analysis and development framework [13]. See online supplementary data appendix 1 for brief descriptions of policy frameworks. While the review did mention the health policy triangle (HPT) framework as a means to help organise and think about the descriptive analysis of key variable types, and to facilitate use of said information in one of the aforementioned political science theories/models, it did not investigate its application to public health policies.

**Fig. 1:**
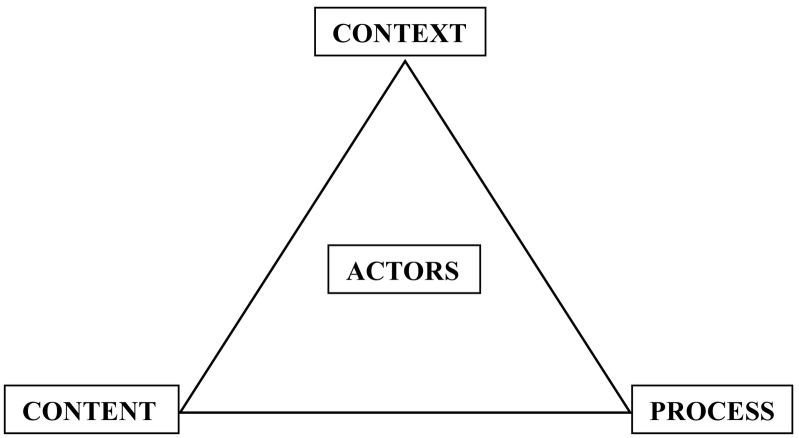
Walt and Gilson policy triangle framework [14].

The HPT framework was designed in 1994 by Walt and Gilson for the analysis of health sector policies, although its relevance extends beyond this sector [14]. They noted that health policy research focused largely on the content of policy, neglecting actors, context and processes (Fig. 1). Content includes policy objectives, operational policies, legislation, regulations, guidelines, etc. Actors refer to influential individuals, groups and organisations. Context refers to systemic factors: social, economic, political, cultural, and other environmental conditions. Process refers to the way in which policies are initiated, developed or formulated, negotiated, communicated, implemented and evaluated [2]. The framework, which can be used retrospectively and prospectively, has influenced health policy research in many countries with diverse systems and has been used to analyse a large number of health issues [15].

In 2015, a historic new sustainable development agenda was unanimously adopted by 193 United Nations (UN) members [16]. World leaders agreed to 17 sustainable development goals (SDGs). These goals have the power to create a better world by 2030; they strive to end poverty, fight inequality and address the urgency of climate change. The SDGs call on all sectors of society to mobilise for action at a global, local and people level. Given that an estimated 40·5 million of the 56·9 million worldwide deaths were from non-communicable diseases in 2016 [17]; approximately 810 women died every day from preventable causes related to pregnancy and childbirth in 2017 [16]; an estimated 6.2 million children and adolescents under 15 years of age died mostly from preventable causes in 2018 [16]; and approximately 38 million people globally were living with HIV in 2019 [16], SDG no. 3 aims to address these issues by ensuring healthy lives and promoting wellbeing for all [16]. This goal has many sub-targets: to reduce maternal mortality; fight communicable diseases; end all preventable deaths under five years of age; promote mental health; achieve universal health coverage (UHC); increase universal access to sexual and reproductive care, family planning and education; and many more. Fortunately, these health topics are regularly examined in the health policy literature and frequently analysed with policy frameworks like the policy triangle model [[18], [19], [20], [21]].

Having established prominence in its field, the objective of this review is to explore and summarise the application of the HPT framework to health-related (public) policy decisions in the recent literature i.e. from January 2015 (corresponding with the year that the SDGs were launched) to January 2020. By investigating the application of the HPT framework to health policies during this time period, such analysis can inform action to strengthen future global policy growth and implementation in line with SDG no.3, and provide a basis for the development of policy analysis work. A review of past literature has previously reported on the wide-ranging use of the HPT framework to understand many policy experiences in multiple lower-middle-income country (LMIC) settings only [15]. This is the first literature review to include a compilation of health policy analysis studies using the HPT framework in both LMIC and high-income country (HIC) settings.

## 2 Methods

### 2.1 Literature search

The Medline, CINAHL Plus with Full Text, Web of Science (Core Collection), APA PsycInfo, PubMed and Embase databases were searched for primary, original literature in English published between 1st January 2015 and 31st January 2020. No Geofilter was applied to the searches. Given the subtle differences which exist between Medline and PubMed databases, it was deemed prudent to search both.

A search strategy was developed based on the use of index and free-text terms related to (i) Health Policy Triangle OR (ii) Policy Triangle Framework OR (iii) Policy Triangle Model. The lack of index terms to describe the HPT framework complicated the development of the search strategy. After much debate and perusal of the literature [9,22], a qualified medical librarian reviewed and approved a search strategy prior to undertaking the literature searches. The search strategy was pre-tested prior to use to maximise sensitivity and specificity and to optimise the difference between both. See online supplementary data appendix 2 for the complete search strategy which attempted to include medical subject headings (MeSH) and Emtree terms and the use of Boolean operators.

**Fig. 2:**
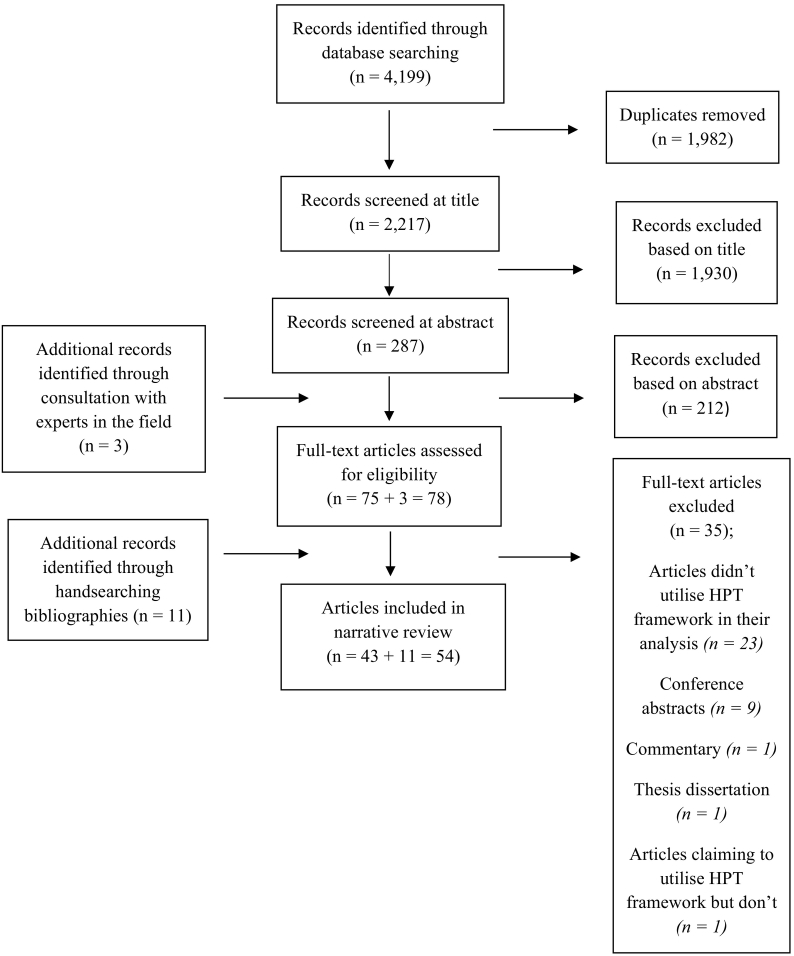
Flow chart of study selection process.

Search results from multiple databases were transferred to a reference manager, End Note X9 [23]. Due to the broad remit of the search strategy, a ‘*title revie*w’ stage was conducted to remove non-pertinent studies (Fig. 2). Studies were removed in a cautious manner. An abstract review was then performed whereupon studies which clearly did not meet the inclusion criteria were excluded. The remaining studies underwent full-text review. To ensure consistency, one reviewer performed all stages of the review. Experts in academia were contacted to provide several suggestions for potentially pertinent studies. A ‘*snowballing*’ approach was used to identify additional literature through manual screening of the reference lists of the retrieved literature as well as the reference lists of such articles eligible for inclusion.

### 2.2 Study selection

The retrieved literature was screened for eligibility according to pre-specified inclusion and exclusion criteria (Table 1).

**Table 1:** Inclusion and exclusion criteria.

Inclusion criteria	Exclusion criteria
(i) Original primary research articles published in English between January 1st, 2015 and January 31st, 2020	(i) Articles not specifically related to health-related/public health policy issues
(ii) Articles interested in the application of the HPT framework to health-related/public health policy issues from countries of all income levels	(ii) Commentaries, conference abstracts, editorials, posters, (research/study) protocols, reports, and white papers
(iii) Articles addressing all four components of the HPT framework i.e. content of the policy; actors involved; process of policy development and implementation; context within which policy is developed	(iii) Book (chapters), (thesis) dissertations and grey literature

### 2.3 Study appraisal and data synthesis

The findings of each study included could not be pooled or combined as in systematic reviews or meta-analyses, and it was not deemed necessary to formally assess the study quality [24]. Indeed, due to the nature of this review, not all of the Preferred Reporting Items for Systematic Reviews and Meta-Analyses (PRISMA) guidelines were relevant, however, insofar as was practical; the PRISMA guidelines were followed [25]. Instead, data from each study included in the review were extracted following guidance from similar studies [9,24,26,27], the National Institute for Health and Care Excellence (NICE) [27] and from the Centre for Reviews and Dissemination's guidance for undertaking reviews in healthcare [28]. Data were extracted and categorised according to country, country classification by income in 2020 [29], study design, data collection method, type and number of participants, type of analysis and health policy field i.e. non-communicable diseases, mental health, tobacco control, etc. The health policy field of the included studies was grouped according to similarity by applying the principles of thematic analysis [30,31]. Occasionally, ambiguity arose as to whether some of the included articles' content concerned health-related/public health policy issues, particularly in relation to the studies which investigated road traffic injury prevention [32] and domestic violence prevention and control [33]. In such instances, a decision of eligibility for inclusion was made after consultation with a co-author.

## 3 Results

### 3.1 Search results

From the literature searches conducted in the six databases, a total of 2217 citations were retrieved after the removal of duplicates. Based upon the title and abstract screening of the citations, 2142 articles were excluded. Another 35 articles were excluded after reading the full texts. Considering the additional records identified through consultation with experts in the field and by handsearching bibliographies, a total of 54 studies were eligible for inclusion in the review. The process of study selection and reasons for exclusions are outlined in Fig. 2. Corresponding authors of all conference abstracts (*n* = 9) excluded were emailed to inquire whether a full-length manuscript of their work was published. The response rate was 100%. As of May 2020, no conference abstract had been published as a full-length manuscript.

### 3.2 Study characteristics

The characteristics of the 54 studies included in the review are summarised in Table 2. Forty-two of these studies describe themselves as having primarily used a qualitative study design. Data collection via various interview formats seemed to be the most common means of information retrieval. Eight of these studies would consider themselves to have a document analysis study design where one of the eight studies also included field work in its methodology. The remaining four studies can be described as respectively having a scoping review, mixed methods approach, literature review and theoretical analysis study design. According to country classification by income in 2020 [29], four of the included studies investigated low-income countries (LICs), 20 LMICs, 16 upper-middle income countries (UMICs), and six HICs. Eight studies were classed as ‘*varied*’ due to multiple countries of different classifications of income being simultaneously examined. All the included studies can be described as some variant of policy analysis. Certain articles highlighted whether the policy analysis was retrospective, prospective or comparative in nature; approximately 20% of the studies incorporated additional conceptual frameworks. Such additional details are outlined in the ‘*Type of analysis*’ column in Table 2. Six studies conducted a supplementary stakeholder analysis/mapping [34].

**Table 2:** Characteristics of included studies (listed alphabetically according to first author).

Study, year	Country	Country classification by income in 2020 [29]	Study design	Data collection	Participants, (n)	Type of analysis	Health policy field
Abiona et al. [35], 2019	Nigeria	LMIC	Qualitative and scoping review	Key informant interviews, document and literature searches	Policy actors and bureaucrats, (*n* = 44) Documents, (*n* = 13)	Policy analysis	Alcohol-related policies
Abolhassani et al. [36], 2017	Iran	UMIC	Qualitative	Semi-structured interviews and document searches	Key informants, (*n* = 31)	Policy analysis including stakeholder analysis	Medication safety policy to restrict look-alike medication names
Akgul et al. [37], 2017	Turkey	UMIC	Qualitative and literature review	Informal interviews, document and literature searches	Key actors, (n =?)	Retrospective policy analysis	Illegal drug policies
Alostad et al. [38], 2019	Bahrain and Kuwait	HIC and HIC	Qualitative	Semi-structured interviews, document searches and direct observation	Key officials, (*n* = 23)	Policy analysis	Herbal medicine registration and regulation
Ansari et al. [39], 2018	Iran	UMIC	Qualitative	Semi-structured interviews	Stakeholders, (*n* = 22)	Policy analysis	Palliative care policymaking
Assan et al. [40], 2019	Ghana	LMIC	Qualitative	Semi-structured interviews	Participants, (*n* = 67)	Policy analysis	Challenges to achieving UHC through community-based health planning and services delivery approach
Azami-Aghdash et al. [32], 2017	Iran	UMIC	Qualitative and literature review	Semi-structured interviews, document and literature searches	Stakeholders, (*n* = 42)	Policy analysis	Road traffic injury prevention
Chen et al. [41], 2019	China	UMIC	Qualitative	Semi-structured interviews and document searches	Key actors, (*n* = 15)	Policy analysis including stakeholder analysis	HPV vaccination programme
Doshmangir et al. [22], 2019	Iran	UMIC	Qualitative	Semi-structured interviews, document analysis and round-table discussion	Stakeholders, (*n* = 23) Round-table discussion (constituting of senior policy makers, *n* = 12)	Policy analysis (HPT incorporating the stages heuristic model)	UHC facilitation in primary healthcare
Dussault et al. [42], 2016	Indonesia, Sudan and Tanzania	LMIC, LMIC and LIC	Field work and document analysis	Field research, document and literature searches	Direct contacts with relevant ministries and agencies, (n = 5) Documents, (n =?)	Policy analysis	Implementation of the health workforce commitments announced at the third global forum on HRH
Etiaba et al. [43], 2015	Nigeria	LMIC	Qualitative and document review	In-depth interviews and document searches	Policy actors, (n = 9)	Retrospective policy analysis	Oral health policy
Faraji et al. [44], 2015	Iran	UMIC	Document analysis	Document searches	Documents, (*n* = 21)	Retrospective policy analysis	Diabetes prevention and control
Guo et al. [45], 2019	China	UMIC	Qualitative	Semi-structured interviews and document analysis	Key actors, (n = 3)	Retrospective policy analysis	National adolescent mental health policy
Hafizan et al. [46], 2018	India, Thailand and Turkey	LMIC, UMIC and UMIC	Scoping review	Journal, article, report and book searches	Articles, (*n* = 26)	Comparative policy analysis	Medical tourism policy
Hansen et al. [47], 2017	Denmark	HIC	Literature review	Journal, article, newspaper and website searches	Articles, (*n* = 11) Newspaper (*n* = 14)	Prospective policy analysis (Kingdon model utilised in addition to HPT)^a^	Implementation of out-of-pocket payments to GPs
Islam et al. [48], 2018	Bangladesh	LMIC	Qualitative	In-depth interviews and document searches	Stakeholders, (*n* = 42)	Policy analysis	Contracting-out urban primary health care
Joarder et al. [49], 2018	Bangladesh	LMIC	Qualitative and literature review	Key informant interviews, document and literature searches	Policy elites, (*n* = 11)	Policy analysis including stakeholder analysis and mapping	Doctor retention in rural settings
Juma et al. [50], 2015	Kenya	LMIC	Qualitative and documents review	Semi-structured interviews and document searches	Stakeholders, (*n* = 19) Documents, (*n* = 14)	Retrospective policy analysis	Integrated community case management for childhood illness
Juma et al. [51,52], 2018	Cameroon, Kenya, Malawi, Nigeria and South Africa	Varied	Qualitative and documents review	Key informant interviews and document searches	Decision-makers, (*n* = 202) Documents, (*n* = 276)	Policy analysis^b^	Multi-sectoral action in non-communicable disease prevention policy development and processes
Kaldor et al. [53], 2018	South Africa	UMIC	Qualitative	Semi-structured interviews	Stakeholders, (n = 10)	Policy analysis	Regulation to limit salt intake and prevent non-communicable diseases
Khim et al. [54], 2017	Cambodia	LMIC	Qualitative and literature review	Key informant interviews, document and literature searches	Participants, (*n* = 29) Documents, (n =?)	Policy analysis	Contracting of health services policy
Le et al. [33], 2019	Vietnam	LMIC	Qualitative and documents review	Key informant interviews and document searches	Policy actors, (*n* = 36) Focus groups, (n = 4) Documents, (*n* = 63)	Policy analysis	Domestic violence prevention and control
Ma et al. [55], 2015	China	UMIC	Qualitative and literature review	In-depth interviews, document and literature searches	Key actors, (*n* = 30) Focus groups, (*n* = 15) Documents, (*n* = 95)	Policy analysis	Task shifting of HIV/AIDS case management to community health service centres
Mambulu-Chikankheni et al. [56], 2018	South Africa	UMIC	Qualitative and document review	In-depth interviews and document searches	Stakeholders, (n = 15) Patient records, (n = 20)	Policy analysis	Role of community health workers in malnutrition management
Mapa-Tassou et al. [57], 2018	Cameroon	LMIC	Qualitative and document review	In-depth interviews and document searches	Stakeholders, (*n* = 38) Documents, (*n* = 19)	Policy analysis	Tobacco prevention and control policies
Mbachu et al. [58], 2016	Nigeria	LMIC	Qualitative and document review	In-depth interviews and document searches	Key informants, (*n* = 10) Documents, (*n* = 5)	Retrospective policy analysis	Integrated maternal newborn and child health
McNamara et al. [59], 2017	Trans-Pacific countries	Varied	Document analysis	Document search(es)	Documents, (n = 1)	Prospective policy analysis (EMCONET framework used in addition to HPT)^c^	Trans-Pacific partnership agreement and associated potentially serious health risks
Misfeldt et al. [60], 2017	Canada	HIC	Qualitative and document review	Key informant interviews and document searches	Stakeholders, (*n* = 30) Documents, (*n* = 119)	Comparative policy analysis	Team-based primary healthcare policies
Mohamed et al. [61], 2018	Kenya	LMIC	Qualitative and document review	Key informant interviews and document searches	Participants, (*n* = 39) Documents, (*n* = 24)	Policy analysis	Formulation and implementation of tobacco control policies
Mohseni et al. [62], 2019	Iran	UMIC	Qualitative and documents review	Semi-structured interviews and document searches	Informants and policymakers, (*n* = 25)	Policy analysis (Kingdon model utilised in addition to HPT)	Prevention of malnutrition among children under five years of age
Mokitimi et al. [63], 2018	South Africa	UMIC	Document analysis	Document searches	Documents, (*n* = 10)	Policy analysis	Child and adolescent mental health policy
Moshiri et al. [64], 2015	Iran	UMIC	Qualitative and literature review	Semi-structured interviews document and literature searches	Key participants, (*n* = 35)	Policy analysis (Kingdon model utilised in addition to HPT)	Formation of primary health care in rural Iran in the 1980s
Mukanu et al. [65], 2017	Zambia	LMIC	Qualitative and document review	Key informant interviews and document searches	Stakeholders, (*n* = 8) Documents, (n = 6)	Policy analysis	Non-communicable diseases policy response
Munabi-Babigumira et al. [66], 2019	Uganda	LIC	Qualitative and document review	In-depth interviews and document searches	Key informants, (*n* = 18)	Policy analysis	Skilled birth attendance policy implementation
Mureithi et al. [67], 2018	South Africa	UMIC	Qualitative and documents review	Key informant interviews and document searches	Participants, (*n* = 56) Focus groups, (*n* = 3)	Policy analysis (Liu's conceptual framework used in addition to HPT)^d^	Emergence of three GP contracting-in models
Mwagomba et al. [68], 2018	Malawi	LIC	Qualitative and document review	Semi-structured interviews and document searches	Key informants, (*n* = 32) Documents, (n = 12)	Policy analysis	Multi-sectoral action in the development of alcohol policies
Nogueira-Jr et al. [69], 2018	Brazil, Chile, Israel	UMIC, HIC, HIC	Qualitative and document analysis	Non-structured interviews, observations and document searches	National team members, (n =?)	Policy analysis^e^	Implementation of national programmes for the prevention and control of healthcare associated infections
O'Connell et al. [70], 2018	Australia, Canada, Ireland, Scotland, Wales	All HIC countries	Document analysis	Document searches	Documents, (n = 8)	Comparative Policy analysis	Frameworks to improve self-management support for chronic diseases
Odoch et al. [71], 2015	Uganda	LIC	Document analysis	Document searches	Documents, (*n* = 153)	Policy analysis (other framework used in addition to HPT)^f^	Male circumcision for HIV prevention policy process
Ohannessian et al. [72], 2018	France	HIC	Document and literature review	Document and literature searches	Documents, (n =?) Articles, (*n* = 4)	Retrospective policy analysis	Non-implementation of HPV vaccination coverage in the pay for performance scheme
Oladepo et al. [73], 2018	Nigeria	LMIC	Qualitative and document review	Key informant interviews and document searches	Stakeholders, (*n* = 44) Documents, (n = 18)	Policy analysis (other framework used in addition to HPT)^g^	Development and application of multi-sectoral action of tobacco control policies
Reeve et al. [74], 2018	Philippines	LMIC	Qualitative and literature review	Semi-structured interviews document and literature searches	Key informants, (n = 21)	Policy analysis (components of ACF and Kingdon model utilised in addition to HPT)	School food policy development and implementation
Roy et al. [75], 2019	India	LMIC	Qualitative and document review	In-depth interviews and document searches	Key stakeholders, (n = 11) Documents, (*n* = 6)	Policy analysis including stakeholder analysis	Adolescent mental health policy
Saito et al. [76], 2015	Laos	LMIC	Qualitative and documents review	Key informant interviews and document searches	Policy implementers, (*n* = 20)	Policy analysis	National school health policy implementation
Shiroya et al. [77], 2019	Kenya	LMIC	Qualitative and documents review	Key informant interviews and document searches	Policy stakeholders, (*n* = 6) Documents, (*n* = 32)	Policy analysis	Translation of the UN declaration to national policies for diabetes prevention and control
Srivastava et al. [78], 2018	India	LMIC	Document and literature review	Document and literature searches	Documents, (*n* = 22)	Retrospective policy analysis	Person-centered care in maternal and newborn health, family planning and abortion policies
Tokar et al. [79], 2019	Ukraine	LMIC	Qualitative and document review	Semi-structured interviews and document searches	Key stakeholders, (*n* = 19) Documents, (*n* = 75)	Policy analysis (other framework used in addition to HPT)^h^	HIV testing policies among female sex workers
Van de Pas et al. [80], 2019	Guinea	LIC	Mixed-methods approach	Semi-structured interviews and quantitative data collection	Key actors, (*n* = 57)	Prospective policy analysis	Health workforce development and retention post-Ebola outbreak
Van de Pas et al. [81], 2017	57 countries and 27 other entities	Varied	Qualitative and literature review	Semi-structured interviews document and literature searches	Government representatives from different countries, (n = 25)	Policy analysis	Implementation of the HRH commitments announced at the third global forum on HRH
Vos et al. [82], 2016	Netherlands	HIC	Qualitative and document analysis	Semi-structured interviews and document searches	Key stakeholders, (n = 12) Documents, (*n* = 64)	Policy analysis including stakeholder analysis	Improvement of perinatal mortality
Wisdom et al. [83], 2018	Cameroon, Kenya, Nigeria, Malawi, South Africa, and Togo	Varied	Qualitative and documents review	Key informant interviews and document searches	Participants, (n = 202) Documents, (n =?)	Policy analysis^i^	Influence of the WHO framework convention on tobacco control on tobacco legislation and policies
Witter et al. [84], 2016	Cambodia, Sierra Leone, Uganda and Zimbabwe	LMIC, LIC, LIC and LMIC	Qualitative and documents review	Key informant interviews and document searches	Participants, (*n* = 109) Documents, (*n* = 270)	Comparative policy analysis including stakeholder mapping	Patterns and drivers of HRH policy-making in post-conflict and post-crisis health systems
Zhu et al. [85], 2018	China	UMIC	Qualitative and literature review	Semi-structured interviews, document and literature searches	Senior policy makers, (n = 2)	Policy analysis^j^	Progress of midwifery-related policies
Zupanets et al. [86], 2018	Ukraine	LMIC	Theoretical analysis	Document and literature searches	Documents, (n =?)	Policy analysis^k^	Development of theoretical approaches to pharmaceutical care improvement and health system integration

Abbreviations: ACF - Advocacy Coalition Framework; AIDS - Acquired Immune Deficiency Syndrome; EMCONET - Employment and Working Conditions Knowledge Network; GP - General Practitioner/Physician; HIC - High-Income Country; HIV - Human Immunodeficiency Virus; HPT – Health Policy Triangle (Framework); HPV – Human Papillomavirus; HRH - Human Resources for Health; LIC - Low-Income Country; LMIC - Lower-Middle-Income Country; UHC – Universal Health Coverage; UMIC - Upper-Middle-Income Country; UN – United Nations; WHO – World Health Organisation; ? – Not specifically mentioned in related text.

a Hansen et al. [47], 2017 - Content and process factors omitted in HPT analysis but justified elsewhere in manuscript.

b Juma et al. [51,52], 2018 - Juma et al. have published two study papers on a related topic from the same project using the same retrieved data sources. Thus, given the similarity, one data entry was deemed sufficient to encompass these two related study papers.

c McNamara et al. [59], 2017 - A framework by the EMCONET of the WHO's Commission on the Social Determinants of Health that comprehensively outlines pathways to health via labour markets [87].

d Mureithi et al. [67], 2018 - A conceptual framework by Liu et al. [88] on the impact of ‘*contracting-out*’ on health system performance.

e Nogueira-Jr et al. [89], 2018 – Actor factor omitted in HPT analysis but justified elsewhere in manuscript.

f Odoch et al. [71], 2015 – Bespoke frameworks used that were conceived from Walt and Gilson's concepts for analysing the inter-relationships between actors, process, and contexts [14]. Odoch et al. also cited Kingdon's multiple stream theory model [12], Foucault's concept of power [90] and the Glassman et al. [91] concept of position mapping of actors, in their bespoke frameworks.

g Oladepo et al. [73], 2018 - Interview guides were informed by the Walt and Gilson policy analysis framework [14] and the McQueen analytical framework for inter-sectoral action [92].

h Tokar et al. [79], 2019 - A framework analysis initially developed by Goffman et al. [93] and adapted by Caldwell et al. [94] was used in order to examine how the HIV/AIDS programme was conceptualised.

i Wisdom et al. [83], 2018 – Wisdom et al. use the same key informant interviews data source that was utilised by Juma et al. [51,52].

j Zhu et al. [85], 2018 – Authors purport to use a policy triangle framework proposed by Hawkes et al. [95]. Upon further inspection and email contact with Hawkes, the framework used was in fact the HPT model originally proposed by Walt and Gilson [14] thus this study was included in the review. It is assumed that the authors accidentally miscited the policy triangle framework in their study.

k Zupanets et al. [86], 2018 – It is unclear which genre of study design best describes this article. For the purposes of this review, its study design was dubbed as a ‘*theoretical analysis*’.

### 3.3 Study findings

From the content analysis approach to the health policy fields of the included studies, five broad descriptive categorised themes were identified demonstrating how the HPT framework was applied to health-related (public) policy decisions in the recent literature: (i) health human resources, services and systems, (ii) communicable and non-communicable diseases, (iii) physical and mental health, (iv) antenatal and postnatal care and (v) miscellaneous. Unsurprisingly, many of the health policy fields explored in the included studies aimed to address sub-targets of SDG no. 3 [16].

#### 3.3.1 Health human resources, services and systems

The implementation of the human resources for health (HRH) commitments announced at the third global forum on HRH [96], with particular attention given to health workforce commitments, were analysed by two separate studies for different countries [42,81]. Another study by Witter et al. focused on the patterns and drivers of HRH policy-making in post-conflict and post-crisis health systems: namely those of Cambodia, Sierra Leone Uganda and Zimbabwe, all lower to lower middle-income countries. Similarly, Van de Pas et al. conducted a policy analysis study which sought to inform capacity development that aimed to strengthen public health systems, and health workforce development and retention, in a post-Ebola LIC setting [80]. Indeed, health workforce retention policy analysis was also carried out by Joarder et al. where retaining doctors in rural areas of Bangladesh was a challenge [49].

Two studies looked at potential issues and policies surrounding UHC facilitation in the primary healthcare setting [22,40]. The somewhat related concept of contracting health services arose in three studies where it was explored in relation to contracting for public healthcare delivery in rural Cambodia [54], contracting-out urban primary healthcare in Bangladesh [48], and the emergence of three general practitioner/physician (GP) contracting-in models in South Africa [67].

At primary and community healthcare level, a variety of policy analysis studies scrutinised topics like the formation of primary healthcare in rural Iran in the 1980s [64], contextual factors and actors that influenced policies on team-based primary healthcare in Canada [60], the potential implementation of out-of-pocket payments to GPs in Denmark [47], and policy resistance surrounding integrated community case management for childhood illness in Kenya [50].

There were three policy analysis studies which focused on medicines and pharmaceutical safety within the health system. Abolhassani et al. reviewed medication safety policy that saw the establishment of the drug naming committee to restrict look-alike medication names [36]. Alostad et al. investigated herbal medicine registration systems policy [38] while Zupanets et al. sought to formulate theoretical approaches to the improvement of pharmaceutical care and health system integration [86].

#### 3.3.2 Communicable and non-communicable diseases

The policy response to non-communicable diseases by the Ministry of Health in Zambia was explored by Mukanu et al. [65], where similarly, Juma et al. investigated non-communicable disease prevention policy development and processes, and how multi-sectoral action is involved [51,52]. Kaldor et al. analysed policy which used regulation to limit salt intake and prevent non-communicable diseases [53]. O'Connell et al. compared frameworks from different countries that aimed to improve self-management support for chronic (non-communicable) diseases [70]. Two studies focused on diabetes, one of the leading non-communicable diseases worldwide, where prevention and control policies for the disease state were reviewed [44,77].

Communicable disease policy analysis studies concentrated on two main viruses; human immunodeficiency virus (HIV) and human papillomavirus (HPV). Analyses in relation to HPV looked at the feasibility of implementation and non-implementation of a HPV vaccination programme in upper-middle to high income countries [41,72]. HIV-related studies varied from policies like task shifting of HIV/AIDS case management to community health service centres [55], and male circumcision for HIV prevention [71], to HIV testing policies among female sex workers [79]. Nogueira-Jr et al. investigated the implementation of national programmes for the prevention and control of healthcare associated infections in three upper-middle to high income countries [69].

#### 3.3.3 Physical and mental health

Alcohol consumption, illegal drugs ingestion, nutritional habits and tobacco inhalation are all potential determinants of the quality of physical health status. Four studies investigated varying factors surrounding tobacco control policies [57,61,73,83]. Two studies examined alcohol-related policies [35,68] where one study scrutinised illegal drug policies [37]. Three studies explored nutrition: two focusing on malnutrition management and prevention in UMICs [56,62] and one reviewing school food policy development and implementation in the Philippines [74]. Interestingly, all three mental health policy analysis studies included in this review focused on the topic of child, and mostly, adolescent mental health policy [45,63,75].

#### 3.3.4 Antenatal and postnatal care

Policy analysis studies regarding pregnancy and mother and child wellbeing featured strongly. Zhu et al. outlined the progress of midwifery-related policies in contemporary and modern China [85] while Munabi-Babigumira et al. analysed the strategies implemented and bottlenecks experienced as Uganda's skilled birth attendance policy was launched [66]. Other studies looked at the various factors which promoted or impeded agenda setting and the formulation of policy regarding perinatal healthcare reform [82], person-centered care in maternal and newborn health, family planning and abortion policies [78], and the integrated maternal newborn and child health strategy [58].

#### 3.3.5 Miscellaneous

There were some other policy analysis studies that can be treated as standalone articles within the context of this review: palliative care system design [39]; national law on domestic violence prevention and control within the health system [33]; oral health policy development [43]; road traffic injury prevention [32]; national school health policy implementation [76]; and medical tourism policy [46]. Interestingly, given that the impact of the Trans-Pacific partnership agreement on employment and working conditions is a major point of contention in broader public debates worldwide [97], one prospective policy analysis study examined the potential health impacts of the Trans-Pacific partnership agreement [98] by investigating labour market pathways [59].

## 4 Discussion

From the findings of this review, the most common method of data collection was by means of some form of interview with participants involved in the relevant policy area. The same finding was found in a similar review [15]. Talking to actors can provide rich information for policy analysis. These collection methods may be the only way to gather valid information on the political interests and resources of relevant actors and to gather historical and contextual information. Indeed, interviews are generally more useful in eliciting information of a more sensitive nature where the goal of the interview is to obtain useful and valid data on stakeholders' perceptions of a given policy issue [2]. However, interview data can be ambiguous in the sense that what interviewees say and the manner in which they say it, may contrast what one actually thinks or does. Many of the studies included in this review overcome this potential limitation by triangulating the responses with additional responses from other informants, or with data collected via alternative channels, particularly documentary sources.

Many different types of policy fields were unearthed throughout the data extraction process. Quite a lot of the studies reviewed large-scale health policies at national level whether that policy be UHC implementation, infectious disease vaccination programmes, or malnutrition management. Some studies conducted policy analysis at international level investigating areas such as the health impact of the Trans-Pacific partnership agreement, and the implementation of the HRH commitments announced at the third global forum on HRH that involved over fifty countries. Cross-country comparative policy analysis was also common and examined topics like medical tourism, factors of HRH policy-making in post-crisis health systems, and frameworks to improve self-management support for chronic diseases. Indeed, health policy fields explored within the descriptive categorised theme ‘*miscellaneous*’ demonstrated how wide-ranging the applicability of the HPT framework is to a variety of health-related (public) policy decisions. None of the included published literature explored policy analysis of local or regional health-related policy decisions using the HPT framework. Given its generalisable nature, further and perhaps more novel uses of the descriptive policy triangle model could be trialed in a diverse range of health policy decisions made at local and regional level.

Of the policy analysis study countries reviewed, approximately 40% were classified as LMIC settings. In recent years, such work has been incorporated into analysis of LMIC public sector reform experiences [15] thus possibly explaining this relatively high percentage. In addition, a reader recently published by WHO to encourage and deepen health policy analysis work in LMIC settings, which considers how to use health policy analysis prospectively to support health policy change, could explain this high percentage [99]. Interestingly, notwithstanding that work conducted within the field of policy analysis is fairly well-established in the United States and Europe [100,101], only approximately 12% of the policy analysis studies yielded from this review were conducted in HIC settings. This finding is open to many interpretations with one crude deduction being that perhaps policy analysis is currently more common in LMIC settings than in HIC settings. Another possibility is that commissioned policy analysis studies in HIC settings are seldom published in peer-reviewed academic journals. Also, it may be the case that LMIC settings rely on external academics to carry out and publish their health policy analysis studies as a recently published evidence assessment reports that LMICs often have an incomplete and fragmented policy framework for research [102]. Further research is required.

All the included studies in this review can be described as some variant of policy analysis where certain articles specifically stated whether the policy analysis was retrospective, prospective or comparative in nature. In fact, the vast majority of studies can be categorised as analyses *of* policy rather than *for* policy [7]. Most of the studies still seek to assist future policy-making, but are largely descriptive in nature, limiting understanding of policy change processes. Similar findings are found in the literature [15].

The comparative policy analysis studies included often involved more than one country with exception of the analysis by Misfeldt et al. who explored the context and factors shaping team-based primary healthcare policies in three Canadian provinces [60]. Although such comparative studies may introduce further challenges (such as working across multiple languages and cultures, and procuring additional funding), the comparisons between similar (and different) country contexts can help disentangle generalisable effects from country context-specific effects in policy adaptation, evolution and implementation [6].

Six studies conducted a supplementary stakeholder analysis/mapping. Stakeholder analysis can be used to help understand about relevant actors, their intentions, inter-relations, agendas, interests, and the influence or resources they have brought or could bring on decision-making processes during policy development [103]. The use of stakeholder analysis in this review was complemented by other policy analysis approaches as is corroborated by the literature [34].

Interestingly, approximately 20% of the studies in this review applied an additional analytical/theoretical framework. McNamara et al. used a framework by the Employment and Working Conditions Knowledge Network (EMCONET) of the WHO's Commission on the Social Determinants of Health [59] which comprehensively outlines pathways to health via labour markets [87]. Mureithi et al. applied a conceptual framework by Liu et al. on the impact of contracting-out on health system performance [67,88]. Odoch et al. decided to implement many bespoke frameworks [71] that were conceived from Walt and Gilson's concepts for analysing the interrelationships between actors, process, and context [14] as well as citing the Kingdon's multiple stream theory model [12], Foucault's concept of power [90] and the Glassman et al. concept of position mapping of actors [91]. Oladepo et al. utilised the McQueen analytical framework for inter-sectoral action [73,92] while Tokar et al. incorporated a framework analysis that was initially developed by Goffman et al. and subsequently adapted by Caldwell et al. in order to examine how the HIV/AIDS programme in question was conceptualised [79,93,94]. Given that there is a paucity of theoretical and conceptual approaches to analysis of the processes of health policy in LMIC settings [6,104], the need to use multiple bespoke frameworks in the aforementioned recent policy analyses may be a plausible finding. In addition, other research has shown that the Walt and Gilson triangle model ‘*needs to be operationalised and transformed*’ in practice which may suggest that it is not fit for purpose in its primitive state [105]. This could explain why auxiliary frameworks are applied alongside the HPT model in these studies.

Other studies applied the Kingdon model in addition to the HPT framework [47,62,64] where Reeve et al. used components of the ACF, Kingdon model and HPT framework [74]. The policy triangle model is often regarded as being descriptive in nature [9,13] thus supplementation with additional frameworks such as the ACF and Kingdon model can enrich the analysis by making it more explanatory [9]. Doshmangir et al. used a tailored version of the HPT framework incorporating the stages heuristic model to guide data analysis [22]. Like the policy triangle model, the stages heuristic are often characterised as being descriptive in nature [9], thus the aforementioned study provided a highly descriptive policy analysis of UHC facilitation in the primary healthcare setting in Iran. Unfortunately, no single policy framework offers a fully comprehensive description or understanding of the policy process as each model answers somewhat different questions [104,106]. Existing policy frameworks have complementary strengths since policy dynamics are driven by a multiplicity of causal paths [107]. Thus, multiple frameworks can be applied as ‘*tools*’ in order to assess and plan action. However, it is important to discern which frameworks may be better suited for particular scenarios and policy issues [106].

Some of the 23 articles (see Fig. 2) that were excluded from this review for not utilising the policy triangle model used other bespoke and well-known health policy frameworks, with the Kingdon's multiple streams theory being the most common [12]. As previously mentioned, a ‘*snowballing*’ approach was used to identify additional literature through manual screening of the reference lists of the retrieved literature as well as the reference lists of such articles eligible for inclusion. Eleven additional studies were identified from this strategy (Fig. 2) meaning many more were excluded for not meeting the inclusion criteria (Table 1). Such studies were too many to document. However, two articles identified from this process appeared to be quite misleading and thus noteworthy. Onwujekwe et al. described a conceptual model that they used in their policy analysis which was almost identical to the HPT framework [108]. However, as the authors didn't characterise or reference their framework to the policy triangle model or to the work of Walt and Gilson, it was omitted from the review. Similarly, Doshmangir et al. portrayed their results in such a way that correlated to the four components of the HPT framework [109]. While the authors did mention the policy triangle framework as a talking point in their discussion section, they failed to explicitly reference it in their methodology and results paragraphs. This led to the exclusion of their study from the review. It is not known why these studies didn't appropriately reference the utilisation of the HPT framework when its application was apparent. It is possible that more policy analysis studies which exist in the recent literature could be presented in a similarly ambiguous manner.

## 5 Limitations

The included articles were mostly qualitative in nature albeit other study designs were also utilised. Limitations inherent to such study designs may present a bias in the quality of the included articles. Grey literature including reports may have provided important sources of information regarding the application of the HPT framework to health-related (public) policy decisions. However, given the difficulty associated with designing internet search strategies, the heterogenous nature of grey literature documents and the additional time required, it was excluded from the review [110]. It was decided to only include primary English-language published literature on this topic from January 2015 to January 2020. It is recommended that additional reviews of other language literature be conducted in association with a wider time frame. This review does not claim to be a fully comprehensive summary of all policy analysis studies which utilised the HPT framework between 2015 and 2020. Further consultation with additional experts, citation searching methods, and handsearching of key journals may produce more relevant articles for inclusion. However, given that the majority of studies analysed thematically in this review are qualitative in nature, it can be argued that it is not necessary to locate every available study for such purposes [31,111]. In addition, it is known that some of the doctoral theses and unpublished material in the field are already represented within the published literature included here. Sometimes, the components of the HPT framework i.e. actors, content, context, process are described as such in the literature without exclusively referring to the HPT framework itself. Thus, these studies would not have been detected using the search strategy chosen for this review (online appendix 2). Finally, when compared to other research designs (e.g. systematic reviews), narrative reviews of the literature are more susceptible to bias e.g. the included articles were not evaluated for their quality [112].

## 6 Conclusion

This narrative review of the recent literature sought, retrieved and summarised the application of the HPT framework to health-related (public) policy decisions. Based on the findings of the review, it appears that the use of this framework appears to be ubiquitous in the health policy literature where many researchers supplement with additional health policy frameworks to further enhance their analysis. Notwithstanding a previous debate which disputes that there is a dearth of theoretical and conceptual approaches to analysis of the processes of health policy in low and middle-income countries [6,104], this review demonstrates that the shortage of health policy analysis studies now appears to come from high income countries. The finding suggests the need for additional health policy analyses to be conducted in such settings, or if this is already happening, the demand to publish more. In relation to the types of health policies being scrutinised, almost all were positioned at national or international level and primarily concerned public health issues. However, given its universal presence in the literature, and its unique adaptability and generalisability to many varied health policy topics, future research applying the HPT framework to smaller scale health policy decisions being investigated at local and regional levels, could be beneficial.

## Funding

This research project was funded by 10.13039/501100002081Irish Research Council (GOIPG/2016/635). The funders had no part in the design of the review; the collection, analysis, and interpretation of the data; the writing of the manuscript; or the decision to submit the article for publication.

## Ethical approval

Ethical approval was not required.

## Author contributions

Gary L O'Brien (GLOB), Sarah-Jo Sinnott (SJS), Stephen Byrne (SB), Valerie Walshe (VW), and Mark Mulcahy (MM): GLOB was responsible for protocol design, study selection, data extraction, drafting of the manuscript and approval of the final manuscript. GLOB conceived the study idea. GLOB, SJS and SB decided on the database selection. GLOB carried out data collection. GLOB analysed and interpreted the data. GLOB wrote the final manuscript; SJS, MM, VW and SB revised the manuscript. All authors read and approved the final manuscript.

## Declaration of competing interest

The authors have no conflicts of interest to declare.

## Online supplementary data

Supplementary material Image 1
