# Supplementary material for: Health policy triangle framework: Narrative review of the recent literature
Source: Health Policy Open. 2020 Oct 6;1:100016. doi: 10.1016/j.hpopen.2020.100016 (PMC10297800; doi:10.1016/j.hpopen.2020.100016)
Supplement: Supplementary file 1 — Supplementary material [file mmc1.docx]

**Electronic Supplementary Material**

**Appendix 1**

| **Brief Description of Policy Frameworks** | |
| --- | --- |
| **Name** | **Description** |
| Advocacy Coalition Framework | The advocacy coalition framework was designed as an alternative to the stages heuristic; it intentionally avoids a linear description of the policy process [1]. It addresses highly challenging issues in which there are substantial goal conflicts, important technical disputes and multiple actors from several levels of Government [2]. The advocacy coalition framework examines the interaction within a policy subsystem of a small number of advocacy coalitions composed of actors from different institutions sharing similar policy beliefs [2]. The advocacy coalition framework describes three tiers of beliefs: (i) deep core beliefs, (ii) policy core beliefs, (iii) secondary beliefs. |
| Institutional Analysis and Development Framework | The institutional analysis and development framework provides a language, and way of thinking about the means in which different institutions foster collective action. It highlights key insights on institutional, technical, and participatory aspects of collective interventions, or the commons problem, and their resulting effects [3]. At the framework’s core is the ‘*action arena*’. The action arena is composed of an action situation and actors and is used as the unit of analysis and investigation [4]. The action situation refers to a social space where the actors interact, solve the commons problem, and exchange goods and services; the actors are those who participate in the situation [2]. A major advantage of the framework is bringing an institutional perspective to policy analysis, which doesn’t appear to be as present in other frameworks. |
| Kingdon’s Multiple Stream Theory | Kingdon’s multiple stream theory within the policy process focuses on the role of policy ‘*entrepreneurs*’ inside and outside Government who take advantage of agenda setting opportunities ‘*policy windows’* and move items onto the Government’s formal agenda [5]. The model postulates that policy choices are made when the three streams (problem stream, policy stream and politics stream) intersect at pivotal time points ‘*policy windows’* where opportunities can occur spontaneously [6]. When a policy window opens, the policy entrepreneur must immediately seize the opportunity to initiate action. |
| Policy Cube | The non-communicable disease policy cube, developed as part of the PA4NCDs project, brings together three axes to assess the strength of a policy framework to combat diet-related non-communicable diseases: comprehensiveness, effectiveness and equity. The fuller the cube, the more robust the policy framework for the prevention and control of non-communicable diseases [7]. |
| Punctuated Equilibrium Model | Punctuated equilibrium model theorises that the policymaking process is characterised by periods of stability with minimal or incremental policy change, disrupted by bursts of rapid transformation [8]. The concept was initially developed in paleontology to explain sudden bursts of change in the fossil record scattered among longer-term minor changes [9]. Central to the theory are the concepts of the ‘*policy image’* and the ‘*policy venue*’. The model has been used to explain the tendency for policy inactivity and sudden change in health policy issues like drug abuse and pesticide control in the USA [10]. |
| Stages Heuristic | The stages heuristic is the ‘i*dealistic*’ to the policy process [11]. It divides the policy process into a series of five stages: (i) agenda setting, (ii) policy formulation, (iii) policy adoption, (iv) policy implementation, and (v) policy assessment. This model has been widely criticised given that its linear, systematic approach to solving policy problems is rarely found. Nonetheless, it is helpful to think of policymaking occurring in these different stages [5]. |

**Appendix 2**

**Search Terms and Strategy Devised upon Consultation with Medical Librarian:**

*Search Terms:*

Health Policy Triangle

OR

Policy Triangle Framework

OR

Policy Triangle Model

i.e.

(Health AND Policy AND Triangle)

OR

(Policy AND Triangle AND (Model or Framework))

Search Strategy Conducted in early February 2020 (Search Restrictions: English Language only, Time period 1^st^ January 2015 – 31^st^ January 2020)

1. The following is the search strategy used for Medline, CINAHL Plus with Full Text, APA PsycInfo in the EBSCO database:

1 (Health.tw AND Policy.tw AND Triangle.tw)

OR

2 (Policy.tw AND Triangle.tw AND (Model or Framework).tw)

154 for APA PsycINFO, 947 for CINAHL, 762 for Medline

1. The following is the search strategy used for Pubmed:

1 (Health AND Policy AND Triangle) All Fields

OR

2 (Policy AND Triangle AND (Model or Framework)) All Fields

599 for Pubmed

(((((Health) AND Policy) AND Triangle AND ("2015/01/01"[PDat] : "2020/01/31"[PDat]))) OR (((Policy) AND Triangle) AND Framework AND ("2015/01/01"[PDat] : "2020/01/31"[PDat]))) OR (((Policy) AND Triangle) AND Model AND ("2015/01/01"[PDat] : "2020/01/31"[PDat]))

1. The following is the search strategy used for EMBASE:

1 (Health AND Policy AND Triangle) All Fields

OR

2 (Policy AND Triangle AND (Model or Framework)) All Fields

559 for EMBASE

#1 OR #2 OR #3

#3

('policy'/exp OR policy) AND triangle AND ('model'/exp OR model) AND [english]/lim AND [embase]/lim AND [1-1-2015]/sd NOT [1-2-2020]/sd

162*

#2

('policy'/exp OR policy) AND triangle AND ('framework'/exp OR framework) AND [english]/lim AND [embase]/lim AND [1-1-2015]/sd NOT [1-2-2020]/sd

63*

#1

('health'/exp OR health) AND ('policy'/exp OR policy) AND triangle AND [english]/lim AND [embase]/lim AND [1-1-2015]/sd NOT [1-2-2020]/sd

**(D)** The following is the search strategy used for Web of Science:

1 ALL=(Health AND Policy AND Triangle) ALL = All Fields

OR

2 ALL=(Policy AND Triangle AND Model)

OR

3 ALL=(Policy AND Triangle AND Framework)

1 OR 2 OR 3 = 1,178 for Web of Science

# 6

1,178

#5 OR #4 OR #3

Indexes=SCI-EXPANDED, SSCI, A&HCI, CPCI-S, CPCI-SSH, BKCI-S, BKCI-SSH, ESCI, CCR-EXPANDED, IC Timespan=2015-2020

# 5

515

(ALL=(Policy AND Triangle AND Model)) AND LANGUAGE: (English)

Indexes=SCI-EXPANDED, SSCI, A&HCI, CPCI-S, CPCI-SSH, BKCI-S, BKCI-SSH, ESCI, CCR-EXPANDED, IC Timespan=2015-2020

# 4

231

(ALL=(Policy AND Triangle AND Framework)) AND LANGUAGE: (English)

Indexes=SCI-EXPANDED, SSCI, A&HCI, CPCI-S, CPCI-SSH, BKCI-S, BKCI-SSH, ESCI, CCR-EXPANDED, IC Timespan=2015-2020

# 3

873

(ALL=(Health AND Policy AND Triangle)) AND LANGUAGE: (English)

Indexes=SCI-EXPANDED, SSCI, A&HCI, CPCI-S, CPCI-SSH, BKCI-S, BKCI-SSH, ESCI, CCR-EXPANDED, IC Timespan=2015-2020manual

References

[1] Weible CM, Sabatier PA, Jenkins‐Smith HC, Nohrstedt D, Henry AD, DeLeon P. A quarter century of the advocacy coalition framework: An introduction to the special issue. Policy Studies Journal 2011; 39:349-60.

[2] Sabatier P. Theories of the policy process. United States of America: Westview Press, 2007.

[3] Nigussie Z, Tsunekawa A, Haregeweyn N, Adgo E, Cochrane L, Floquet A, Abele S. Applying Ostrom’s institutional analysis and development framework to soil and water conservation activities in north-western Ethiopia. Land Use Pol 2018; 71:1-10.

[4] Moloughney BWP. The Use of Policy Frameworks to Understand Public Health-Related Public Policy Processes: A Literature Review. Peel Public Health, 2012.

[5] Buse K, Mays N, Walt G. Making health policy. McGraw-Hill Education (UK), 2012.

[6] Kingdon JW. Agendas, Alternatives, and Public Policies,(2nd) HarperCollins College Publishers. New York 1995.

[7] Buse K, Aftab W, Akhter S, Phuong LB, Chemli H, Dahal M, Feroz A, Hofiani S, Pradhan NA, Anwar I, Skhiri HA, El Ati J, Giang KB, Puri M, Noormal B, Rabbani F, Hawkes S. The state of diet-related NCD policies in Afghanistan, Bangladesh, Nepal, Pakistan, Tunisia and Vietnam: a comparative assessment that introduces a ‘policy cube’approach. Health Policy and Planning 2020.

[8] Walt G, Shiffman J, Schneider H, Murray SF, Brugha R, Gilson L. ‘Doing’health policy analysis: methodological and conceptual reflections and challenges. Health policy and planning 2008; 23:308-17.

[9] Givel M. The evolution of the theoretical foundations of punctuated equilibrium theory in public policy. Review of Policy Research 2010; 27:187-98.

[10] Baumgartner FR, Jones BD. Agendas and Instability in American Politics. University of Chicago Press, 1993.

[11] DeLeon P. The stages approach to the policy process: What has it done? Where is it going. Theories of the policy process 1999; 1:19-32.
